# Piloting a Deep Learning Model for Predicting Nuclear BAP1 Immunohistochemical Expression of Uveal Melanoma from Hematoxylin-and-Eosin Sections

**DOI:** 10.1167/tvst.9.2.50

**Published:** 2020-09-01

**Authors:** Hongrun Zhang, Helen Kalirai, Amelia Acha-Sagredo, Xiaoyun Yang, Yalin Zheng, Sarah E. Coupland

**Affiliations:** 1 Department of Eye and Vision Science, Institute of Life Course and Medical Sciences, University of Liverpool, Liverpool, UK; 2 Liverpool Ocular Oncology Research Group, Department of Molecular and Clinical Cancer Medicine, Institute of Systems, Molecular and Integrative Biology, University of Liverpool, Liverpool, UK; 3 Liverpool Clinical Laboratories, Liverpool University Hospitals NHS Foundation Trust, Liverpool, UK; 4 Chinese Academy of Sciences (CAS) IntelliCloud Technology Co., Ltd., Shanghai, China

**Keywords:** uveal melanoma, choroidal melanoma, hematoxylin-and-eosin (H&E), BAP1, prognostication, whole slide imaging, deep learning, artificial intelligence

## Abstract

**Background:**

Uveal melanoma (UM) is the most common primary intraocular malignancy in adults. Monosomy 3 and *BAP1* mutation are strong prognostic factors predicting metastatic risk in UM. Nuclear BAP1 (nBAP1) expression is a close immunohistochemical surrogate for both genetic alterations. Not all laboratories perform routine BAP1 immunohistochemistry or genetic testing, and rely mainly on clinical information and anatomic/morphologic analyses for UM prognostication. The purpose of our study was to pilot deep learning (DL) techniques to predict nBAP1 expression on whole slide images (WSIs) of hematoxylin and eosin (H&E) stained UM sections.

**Methods:**

One hundred forty H&E-stained UMs were scanned at 40 × magnification, using commercially available WSI image scanners. The training cohort comprised 66 BAP1^+^ and 74 BAP1^−^ UM, with known chromosome 3 status and clinical outcomes. Nonoverlapping areas of three different dimensions (512 × 512, 1024 × 1024, and 2048 × 2048 pixels) for comparison were extracted from tumor regions in each WSI, and were resized to 256 × 256 pixels. Deep convolutional neural networks (Resnet18 pre-trained on Imagenet) and auto-encoder-decoders (U-Net) were trained to predict nBAP1 expression of these patches. Trained models were tested on the patches cropped from a test cohort of WSIs of 16 BAP1^+^ and 28 BAP1^−^ UM cases.

**Results:**

The trained model with best performance achieved area under the curve values of 0.90 for patches and 0.93 for slides on the test set.

**Conclusions:**

Our results show the effectiveness of DL for predicting nBAP1 expression in UM on the basis of H&E sections only.

**Translational Relevance:**

Our pilot demonstrates a high capacity of artificial intelligence-related techniques for automated prediction on the basis of histomorphology, and may be translatable into routine histology laboratories.

## Introduction

Uveal melanoma (UM) is the most common primary intraocular malignancy in adults.^1^ Although ocular treatments have high rates of success in controlling the tumor locally, approximately 50% of patients develop metastatic disease to the liver.^1^ Disseminated UM is unfortunately, at present, incurable.^2^

Various parameters are well-known for determining patients’ with UM prognosis, and this enables their stratification into metastatic risk groups for surveillance of the liver.^3^^,^^4^ Should the metastases be detected earlier, patients can either undergo liver surgery for removal of metastatic UM nodules, or be registered into clinical trials.^5^ Prognostic parameters for primary UM include clinical features (age and gender of the patient; intraocular tumor location; and size and extent of tumor growth), and both histomorphological and genetic features of the tumor.^1^ With respect to the genetic features, one of the strongest parameters is the status of chromosome 3 in the UM cells: loss of one copy of chromosome 3 (i.e. monosomy 3) is associated with a poor prognosis.^6^ Located on chromosome 3 (3p21.1) is the gene *BAP1 (BRCA1* associated protein-1), which encodes for the deubiquitinating enzyme, ubiquitin carboxy-terminal hydrolase.^7^ *BAP1* mutations are associated with cancers, such as clear cell renal carcinoma, mesothelioma, and non-small cell lung cancer as well as UM.^6^^–^^8^ Somatic inactivating mutations in *BAP1* have been reported in 18 to 48% of all UM, and in approximately 84% of UM with monosomy 3; this is strongly associated with metastasis and poor patient prognosis.^6^^–^^11^ The bi-allelic inactivation of *BAP1* frequently leads to a loss of nuclear BAP1 protein expression (nBAP1) on immunohistochemistry (IHC; i.e. it can be used as a surrogate marker because its nuclear expression corresponds to a high degree with both *BAP1* mutational and chromosome 3 status).^9^^,^^10^

Recent work using digital image analysis (DIA) of enucleated UM specimens stained with BAP1 IHC demonstrated that DIA is a competitive alternative to manual assessment as well as gene expression profiling in prognostication of these tumors.^12^ Because not all laboratories have access to high quality IHC facilities or to genetic testing, DIA and artificial intelligence (AI) has been applied to a variety of cancer types to predict underlying genomic changes from conventional stains.^13^^–^^16^ Deep learning (DL), one of the major branches in AI, has burgeoned over the past decade for its superior performance compared with traditional approaches relying on hand-crafted features.^17^ With the help of DL techniques, we developed a pilot algorithm to predict nBAP1 expression based on hematoxylin and eosin (H&E) sections only of clinically and genetically well-defined cohorts of UM.

## Materials and Methods

### Ethics

This study conformed to the principles of the Declaration of Helsinki and Good Clinical Practice guidelines. Approval for the study was obtained from the Health Research Authority (NRES REC ref 15/SC/0611), and all patients provided informed consent.

### UM Samples

H&E and BAP1-stained sections from 184 patients with UM treated by enucleation or local resection at the Liverpool Ocular Oncology Centre (LOOC), Liverpool University Hospitals National Health Service (NHS) Foundation Trust between January 2013 and December 2015 were included in this study. The specimens were processed with the Liverpool Clinical Laboratories, stained using conventional and immunohistochemical stains (including BAP1, as previously described^9^^,^^10^), and reported by the senior author.

**Figure 1.**
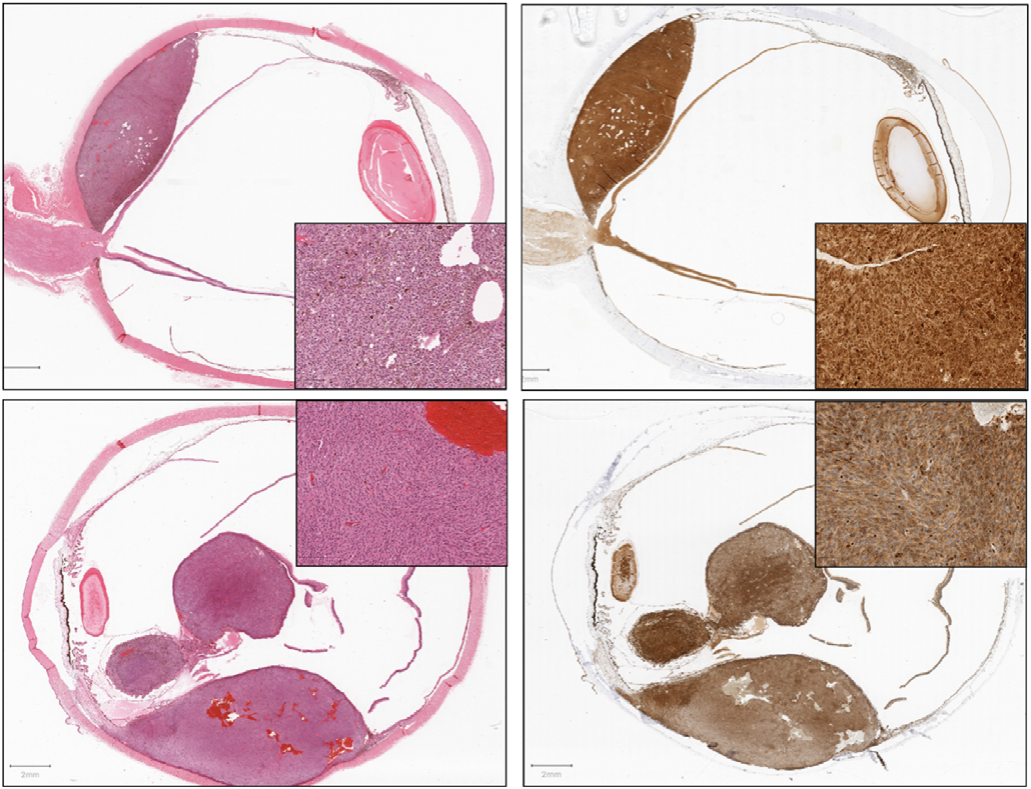
Low- and high-power magnification images of enucleated eyes stained for hematoxylin and eosin (H&E) and BAP1 immunohistochemistry, taken after whole slide scanning. **Top row:** This is a nBAP1 positive UM; **bottom row:** a nBAP1 negative UM. The insets provide the higher power magnification of the tumor morphology and the location of the BAP1 staining.

In total, there were 140 slides used for the training set, including 66 nBAP1^+^ and 74 nBAP1^−^. A second cohort of UM cases (*n* = 14) was retrieved from the Liverpool Clinical Laboratory archive, and used as the *test set* (16 nBAP1^+^ and 28 nBAP1^−^). The scoring of the nBAP1 staining of the training set UM and the association of nBAP1 IHC to tumor cell morphology, molecular genetics, and clinical outcomes has been reported in detail recently.^9^ Chromosome 3 status was available for all patients with UM and was determined, as previously described.^9^ The details of the “training” and “test set” cohorts of UM are provided in Supplementary Tables S1 and S2. Examples of low and high magnification of H&E and BAP1-stained UM are provided in Figure 1.

### Whole Slide Imaging

#### Sample Statistics

The H&E-stained sections of all patients with UM in the training set were scanned at 40 × magnification using Aperio CS2 (LeicaBiosystems, Newcastle-Upon-Tyne, UK), and saved as whole slide images that have extremely high resolution. These slides of the test set were scanned also at 40 × magnification using the VentanaDP200 (Roche, West Sussex, UK). Random slides from both cohorts were also scanned on both platforms. The use of the differing platforms was undertaken to determine the flexibility of the algorithm.

#### Generation of Tumor Patches

**Figure 2.**
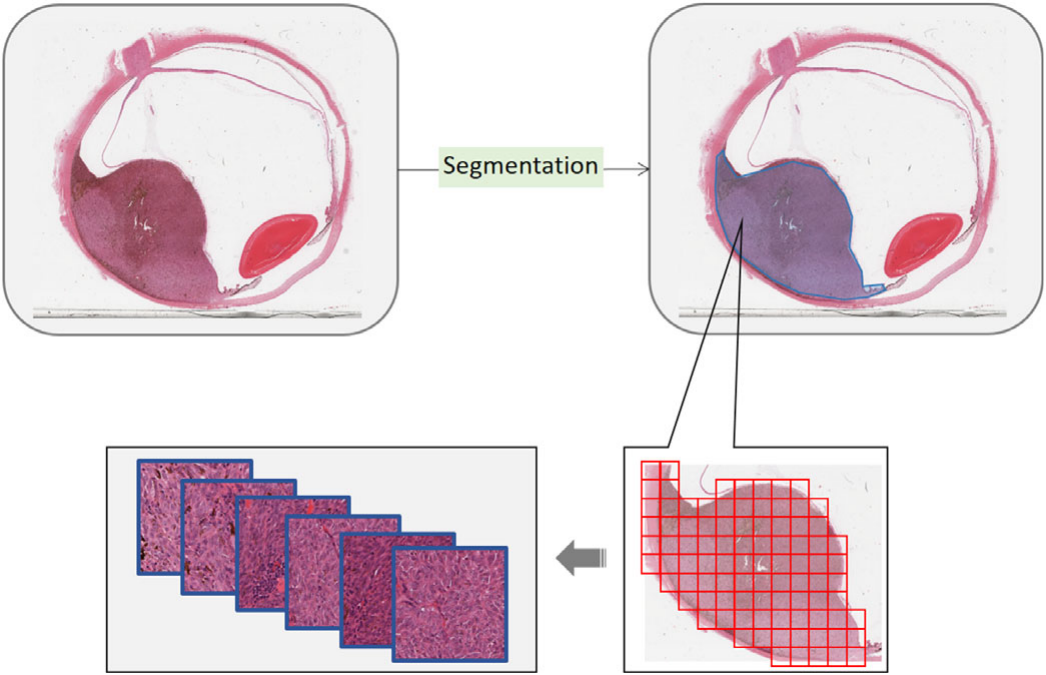
Generation of tumor patches. The tumor region is first segmented from a whole slide image. The tiling operation is then applied to the tumor region. A tile with tumor ratio over 90% is cropped out as a tumor patch.

From the whole slide images (WSIs), UM regions were first recognized visually in the H&E section and “cropped out” (Fig. 2). The nonoverlapping tiling operation was then applied to the cropped tumor regions and patches with > 90% tumor area were selected. By referencing to the counterpart BAP1-stained slide, all the selected patches were labelled on the corresponding H&E section, either as BAP1^+^ or BAP1^−^. From the total 184 UM slides (i.e. both the training and test sets), 539848, 130471, and 30677 tumor patches were cropped in 3 different dimensions, respectively (Table 1).

**Table 1.** Data With Respect to Patch Size and Numbers in the Training and Test Sets

	Training Set (Including Validation Set)		Test Set	
Patch Dimension	Positive	Negative	Positive	Negative
512 × 512	160,059	253,097	49,769	76,923
1024 × 1024	38,589	61,189	12,066	18,627
2048 × 2048	9,007	14,275	2,826	4,569

### DL for Prediction of nBAP1 Expression

**Figure 3.**
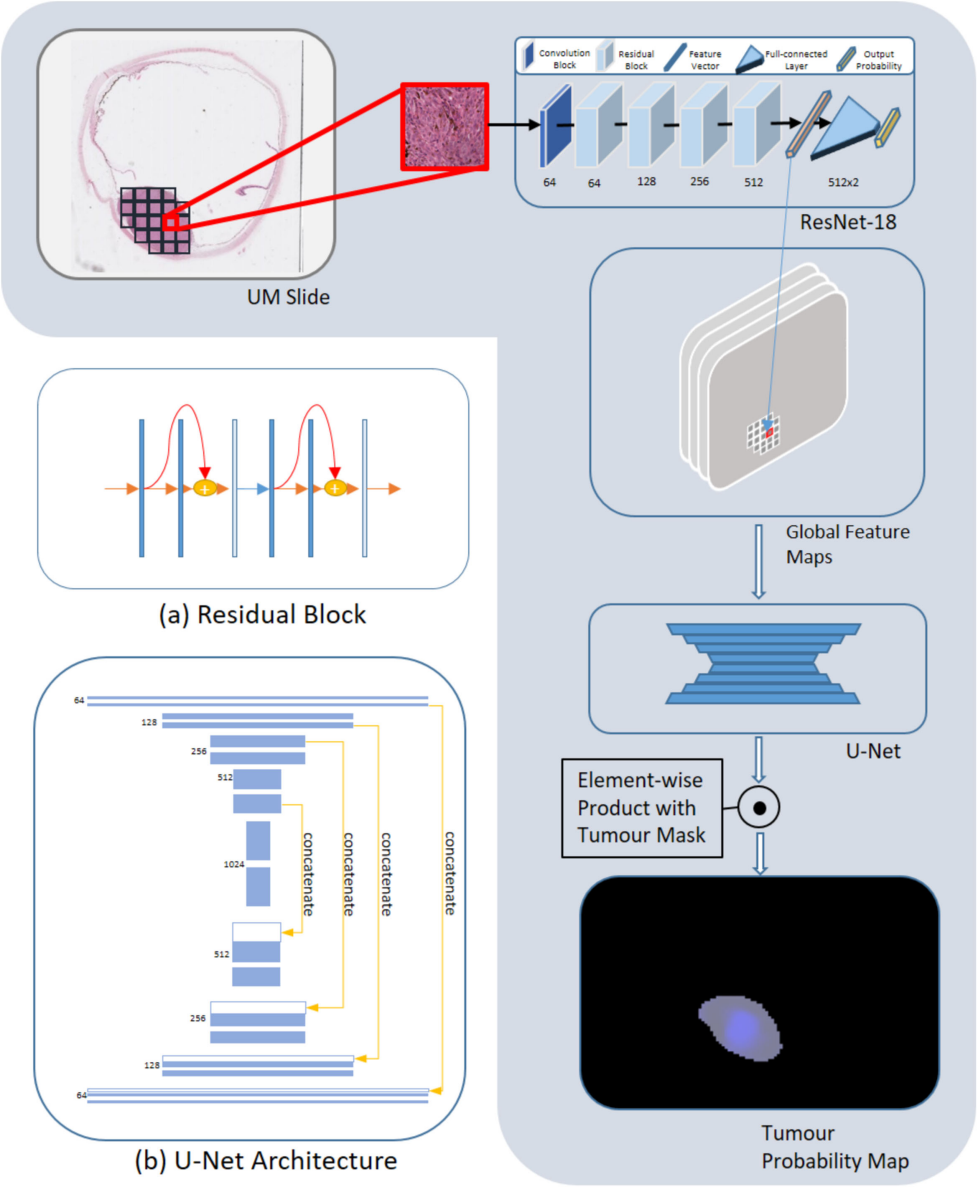
Schematic diagram to show the process to predict patch-level nBAP1 expression. All the tumor patches in a slide are fed to a trained ResNet-18, which outputs the posterior probabilities of the patches (this is referred to independent patch classification). The feature vector corresponding to a tumor patch is extracted from the convolutional module (**a**) in the ResNet-18. The feature vectors of all the tumor patches are re-assembled into global feature maps according to the locations of tumor patches in the slide. The global feature maps are then forward to a U-Net (**b**), that outputs the probability map of the slide. The posterior probabilities of patches can be produced by a region correlation classification from the probability map of the slide. **a** A diagram shows a standard residual block used in the ResNet-18; **b** a diagram represents the U-net architecture used.

A “bottom-up approach” was developed for the prediction of nBAP1 expression using a deep convolutional neural network (DCNN). The DCNNs, as a subgroup of DL networks, are dedicated for image processing tasks,^18^^,^^19^ and can achieve an image classification in an end-to-end manner without the need of handcrafting features from images for classification. In this study, a binary prediction was obtained (i.e. nBAP1^+^ and nBAP1^-^). The overall framework is shown in Figure 3. In brief, by utilizing the labeled patches, a ResNet-18^20^ was trained to predict nBAP1 expression of each patch. The ResNet-18 served not only as a classifier but also as a feature extractor. The extracted feature vectors of all the tumor patches in a slide were re-assembled as a set of feature maps according to their spatial locations, defined as the “global feature” map, and subsequently were fed into an auto-encoder-decoder that outputted the probability maps. By applying the element-wise product with the tumor masks, one element in a probability map corresponding to a tumor patch in the original slide, indicated as the “posterior probability” for this tumor patch to be nBAP1^+^.

#### Independent Patch Prediction

Fine tuning is a widely used approach for transfer learning to train DL models and for image classification tasks, it is a common practice to train application specific models by fine-tuning ImageNet pretrained models for rich features they have learned. A ResNet-18 pretrained on ImageNet^21^ was fine-tuned again to independently classify each individual patch's nBAP1 expression status. The skip connection operation in ResNet-18 concatenates feature maps of a functional convolution module (typically comprising a convolution layer, pooling layer, batch normalization layer, and activation layer consecutively in order) with the feature map (resized if necessary) from a previous module (see Fig. 3a). This mechanism enables back-propagated gradients to be amplified to alleviate the gradient-vanishing effect.^22^ ResNet-18 was chosen based on the tradeoff between the performance and the computational consumption; however, other networks, such as VGG-Nets^23^ or Dense-Nets,^24^ will equally work. To train a ResNet-18 model, weighted cross-entropy was adopted as the loss function to compensate the imbalance between the positive and negative patches. Specifically, the losses from the positive patches were scaled up by a weight that was inversely proportional to the ratio of all positive patches in the training set, and vice versa for negative patches.

#### Tumor Patches Prediction with Region Correlation

Intuitively, a tumor patch is more likely to be nBAP1^+^ if its surrounding tumor patches are also nBAP1^+^, and vice versa. We define this prior information as the “region-correlation.” It has been empirically proven that the region correlation was effective to improve the accuracy of histopathology patches classification.^25^^–^^28^ To explore the effectiveness of region correlation for patch classification, we further imposed region correlation on the global feature to obtain the resultant global probability map. The resultant probability of each tumor patch can be directly read from the global probability map.

We adopted the strategy modified from Takahama et al.^28^ to utilize region information. This allowed us to directly utilize the benefits of well-developed and “off-the-shelf” auto-encoder-decoder models for this purpose. The U-Net architecture^29^ used is characterized by the short-cut connections of feature maps among the counterpart layers from the encoder and decoder modules, respectively (see Fig. 3). Specifically, the feature vector for each patch in a WSI was extracted by the convolutional module in the trained ResNet-18, and then re-organized to form a set of global features maps based on their locations in the slide, which serves as the input to a U-Net model. The U-Net model outputted a probability map with the same dimension as the input global feature maps. An element value in the probability map indicated the posterior probability to be nBAP1^+^ for the corresponding tumor patch in the WSI.

A weighted cross-entropy on the probability maps was used as the loss function to train the U-Net model whereas only the losses corresponding to tumor patches were considered. This was implemented by an element-wise product of the probability maps and the corresponding tumor masks before calculating the weighted cross-entropy. The final decision on BAP1 status of the corresponding H&E-stained whole slide was achieved by averaging the output probability of all the patches in the tumor.

### Experimental Configurations

#### Patch Pre-Processing

The experiments were performed independently on 3 different dimensions of patches cropped from slides of 40 × magnification, namely 512 × 512, 1024 × 1024, and 2048 × 2048, respectively, and the patches of different dimensions were all further resized to 256 × 256. For training purposes, a sub-patch (224 × 224) was randomly cropped from an original patch (256 × 256), and then was randomly flipped horizontally or vertically. Color jitter was applied on the sub-patch for data augmentation before being fed to ResNet-18, which randomly changed the brightness, contrast, saturation, and hue of the sub-patch with ranges all between approximately 0.6 and 1.4. During validation and test periods, no data augmentations were used and sub-patches (224 × 224) were cropped from the center of original patches (256 × 256).

#### Networks Training

All the 140 slides for training were randomly and evenly split into 5 subgroups, with each subgroup having approximately even numbers of nBAP1^+^ and nBAP1^−^ slides. For each dimension of patches, five models (each included a ResNet-18 and a U-Net) were trained, and each used one of the subgroups of slides as the validation dataset, whereas the remaining four subgroups were used as the training dataset (Table 2). During training, the parameters of each model that achieved the best performance on the corresponding validation set were saved and tested on the test set. Importantly, although the predictions were performed on patches, the training dataset, validation set, and test sets were split at the slide-level basis, instead of at the patch-level in order to avoid “information leaking,” based on the intuition that patches from the same slide are highly correlated.

**Table 2.** Different Subsets (S1–S5) in the Training Set for Training and Validation, Respectively

	Subsets for Training	Subset for Validation
Model 1	S2, S3, S4, S5	S1
Model 2	S1, S3, S4, S5	S2
Model 3	S1, S2, S4, S5	S3
Model 4	S1, S2, S3, S5	S4
Model 5	S1, S2, S3, S4	S5

Stochastic gradient descent (SGD) was adopted as the training optimizer with a momentum of 0.9 and weight decay of 0.0005 for both the ResNet and U-Net. A ResNet-18 was trained for 30 epochs with an initial learning rate of 0.001, and the learning rate was divided by 5 at epoch 10 and epoch 20, respectively. A U-Net was trained with a constant learn rate of 0.0001 for 100 epochs.

### Performance Metrics

Area under the receiver operating characteristic curve (AUC) was the main performance metric used to evaluate the trained models. A receiver operating characteristic (ROC) profiles the relationship between the sensitivity and specificity and is obtained by sliding a threshold (between 0 and 1) over the nBAP1^+^ probability to calculate the corresponding sensitivity and specificity values. An ROC closer to the top left corner means better performance; consequently, a better AUC has the value closer to 1. Besides, accuracy, precision, and F1 value were also adopted, which were calculated with the probability threshold 0.5.

## Results

### Patch-Based Prediction

In what follows, we use the notation “model(n)-k” to represent the k_th_ model that trained and tested on the “n” by “n” patches. For example, model (1024)-2 is the second model that trained and tested on the 1024 × 1024 patches.

For independent patch classification (from ResNet-18), the ranges of the AUC values of 5 models are 0.66 to 0.80, 0.80 to 0.84, and 0.77 to 0.81 for patch size of 512 × 512, 1024 × 1024, and 2048 × 2048, respectively. The corresponding mean ± standard deviation (SD) are 0.711 ± 0.059, 0.825 ± 0.012, and 0.805 ± 0.023, respectively. For patch classification with region correlation (from U-Net), the AUC values of 5 models range from 0.67 to 0.80, 0.83 to 0.90, and 0.79 to 0.85 for patch sizes of 512 × 512, 1024 × 1024, and 2048 × 2048, respectively. The corresponding mean ± SD are 0.753 ± 0.055, 0.861 ± 0.026, and 0.823 ± 0.027, respectively. The patch-level performances (AUC) are shown in Table 3.

**Table 3.** Patch-Level Area Under Curve (AUC) on the Test Set

**Patch Dimension:512** **×** **512**						
	**Model (512)-1**	**Model (512)-2**	**Model (512)-3**	**Model (512)-4**	**Model (512)-5**	**Mean/Standard Deviation**
Independent	0.730	0.665	0.676	0.805	0.675	0.710/0.058
	(0.727–0.733)	(0.662–0.668)	(0.673–0.679)	(0.803–0.807)	(0.672–0.678)	
Region correlation	0.804	0.721	0.766	0.797	0.674	0.753/0.054
	(0.802–0.80)	(0.719–0.724)	(0.763–0.769)	(0.794–0.799)	(0.672–0.677)	
**Patch Dimension: 1024** **×** **1024**						
	**Model (1024)-1**	**Model (1024)-2**	**Model (1024)-3**	**Model (1024)-4**	**Model (1024)-5**	**Mean/Standard Deviation**
Independent	0.824	0.809	0.823	0.843	0.823	0.8249/0.011
	(0.819–0.829)	(0.805–0.814)	(0.818–0.828)	(0.839–0.847)	(0.819–0.828)	
Region correlation	0.853	0.837	0.850	0.904	0.858	0.8605/0.025
	(0.849–0.857)	(0.832–0.841)	(0.845–0.854)	(0.900–0.907)	(0.853–0.862)	
**Patch Dimension: 2048** **×** **2048**						
	**Model (2048)-1**	**Model (2048)-2**	**Model (2048)-3**	**Model (2048)-4**	**Model (2048)-5**	**Mean/Standard Deviation**
Independent	0.773	0.788	0.819	0.826	0.817	0.8048/0.022
	(0.762–0.784)	(0.777–0.798)	(0.809–0.828)	(0.816–0.835)	(0.807–0.827)	
Region correlation	0.797	0.793	0.857	0.828	0.840	0.8233/0.027
	(0.786–0.808)	(0.783–0.804)	(0.848–0.866)	(0.818–0.838)	(0.830–0.849)	

The 95% confidence intervals (CIs) are in parentheses.

The best performance was provided by model (1024)-4 with region correlation, that achieved an AUC value of 0.90 (95% confidence interval [CI]: 0.901–0.908). Further, it can be concluded that patches with a size of 1024 × 1024 performs the best, with the corresponding mean ± SD AUCs are 0.825 ± 0.012 (independent patch classification) and 0.861 ± 0.023 (region correlation), respectively. In contrast, patches with a dimension of 512 × 512 performed worst and unstable with smallest mean value and largest among all. By comparison, it also shows that by imposing region correlation, most cases achieved higher AUC values, with improvements that ranged from 2% to 5%, compared with those by independent patch classification.

For comparison, we also re-implemented the method from Sun et al.,^30^ which was developed for the classification of patches from *BAP1*-stained UM slides, and applied it to the our dataset with the same configuration of sample splitting. Table 4 shows the corresponding AUCs. It can be seen that when trained with the same subset of samples, our method outperforms that based on the BAP-1 stained slide,^30^ and that the performance gain can be even up to 5% (from model (1024)-4).

**Table 4.** Patch-Level Area Under Curve (AUC) on the Test Set of the Method from Sun et al.^30^ and Our Method (with Region Correlation)

	Model (1024)-1	Model (1024)-2	Model (1024)-3	Model (1024)-4	Model (1024)-5
Sun et al.^30^	0.846 (0.842–0.850)	0.799 (0.794–0.804)	0.849 (0.844–0.853)	0.854 (0.850–0.859)	0.847 (0.843–0.851)
Our method	0.853 (0.849–0.857)	0.837 (0.832–0.841)	0.850 (0.845–0.854)	0.904 (0.900–0.907)	0.858 (0.853–0.862)

The 95% confidence intervals (CIs) are in parentheses.

#### Ensemble from Five Models

**Figure 4.**
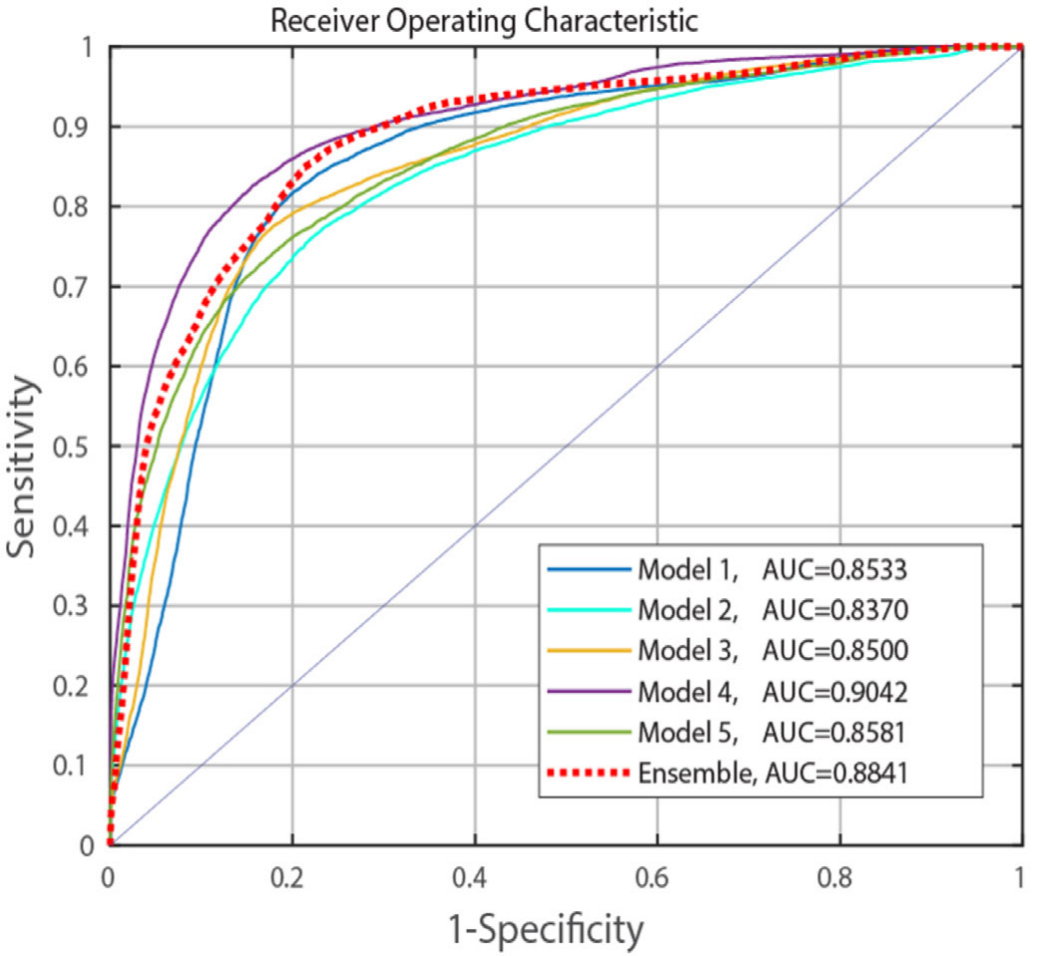
Receiver operating characteristic (ROC) curve for patch classification (Patch dimension: 1024 × 1024).

Giving a patch of a certain dimension, we calculated its “ensemble” posterior probability by averaging the posterior probabilities of this patch from the five models. Table 5 presents the corresponding ensemble results. It shows the results by ensemble can always achieve the best or near best performances among the results from the five network models, as is further confirmed by Figure 4, where the ensemble ROC is the one most close to the best ROC (model (1024)-4) and is above the four curves of other models. The best performance was also on the patches of 1024 × 1024 with an AUC value of 0.884 (95% CI: 0.879, 0.886).

**Table 5.** Patch-Level Area Under Curve (AUC) on the Test Set of the Ensemble Model

Patch Dimension	512 × 512	1024 × 1024	2048 × 2048
Independent	0.753 (0.752, 0.753)	0.849 (0.847, 0.851)	0.823 (0.812, 0.831)
Region-Correlation	0.825 (0.824, 0.826)	0.8841 (0.878, 0.886)	0.8401 (0.835, 0.848)

The 95% confidence intervals (CIs) are in parentheses.

#### Probability Maps

**Figure 5.**
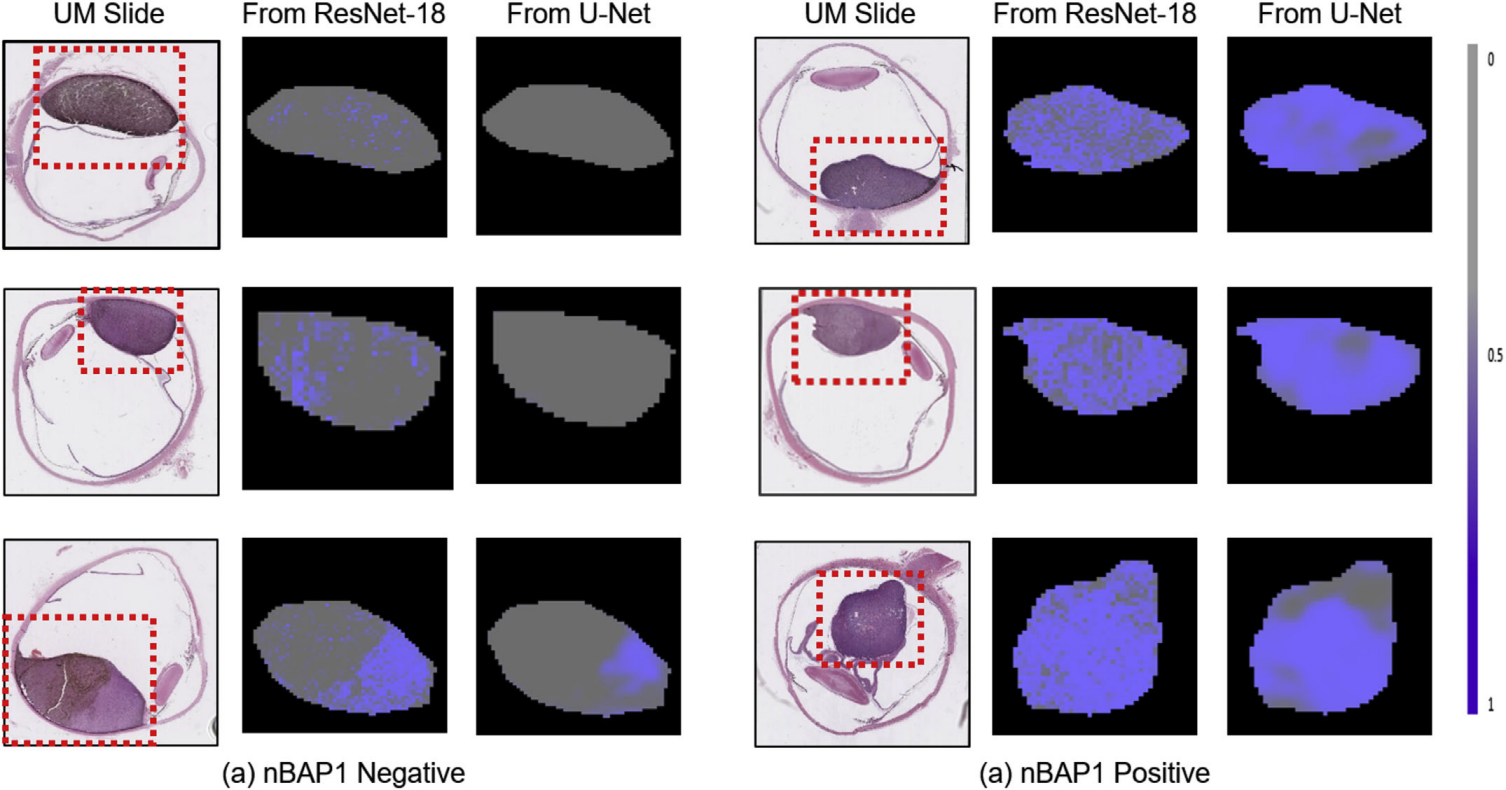
Probability maps. The first column lists the UM slide stained with H&E, and their probability maps by independent patch classification (from ResNet-18) and by region-correlation patch classification (from U-Net) were shown on the second and third columns, respectively. The probability maps corresponding to the red dotted regions in the UM slide with zooming. Grey corresponds to nBAP1 negative, whereas blue corresponds to nBAP1 positive.

Figure 5 presents the probability maps of six randomly selected slides from the test sets. For independent patch classification, the probability maps were generated by stitching the probabilities of the tumor patches from ResNet-18 into all-zero maps, whereas for patch classification with region correlation the probabilities were from the element-wise product of outputs from U-Net and the tumor masks. Clearly, by independent classification of patches, the probabilities are scattering in a probability map. Whereas with region correlation, the probability maps in comparison are more cohesive and consistent locally.

### Classification Results at the Whole Slide Level

The nBAP1 expression of a UM in the same slide is essentially unified (i.e. the BAP1 expression in a slide can either be positive or negative, but cannot have two co-existing states simultaneously). Because of this, it is meaningful to categorize a slide by the BAP1 expression. To this end, we calculated the posterior probability of a slide to be BAP1 positive by averaging the posterior probabilities of all the tumor patches in it. The posterior probabilities of patches can be from a single model or from the “ensemble” of the five individual models.

**Figure 6.**
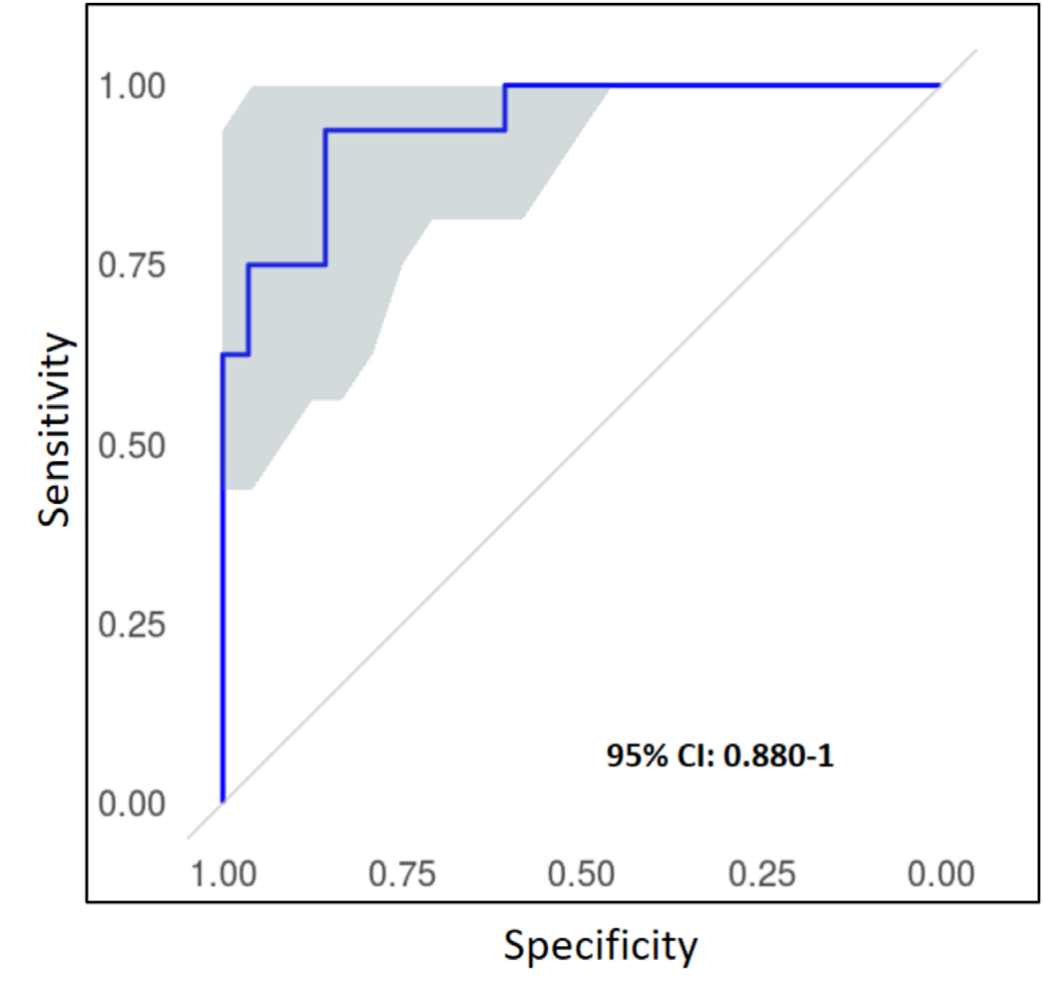
Slide-level receiver operating characteristic (ROC) curve with 95% confidence intervals of the ensemble of 5 models (independent patch classification) trained on 1024 × 1024 patches.

**Figure 7.**
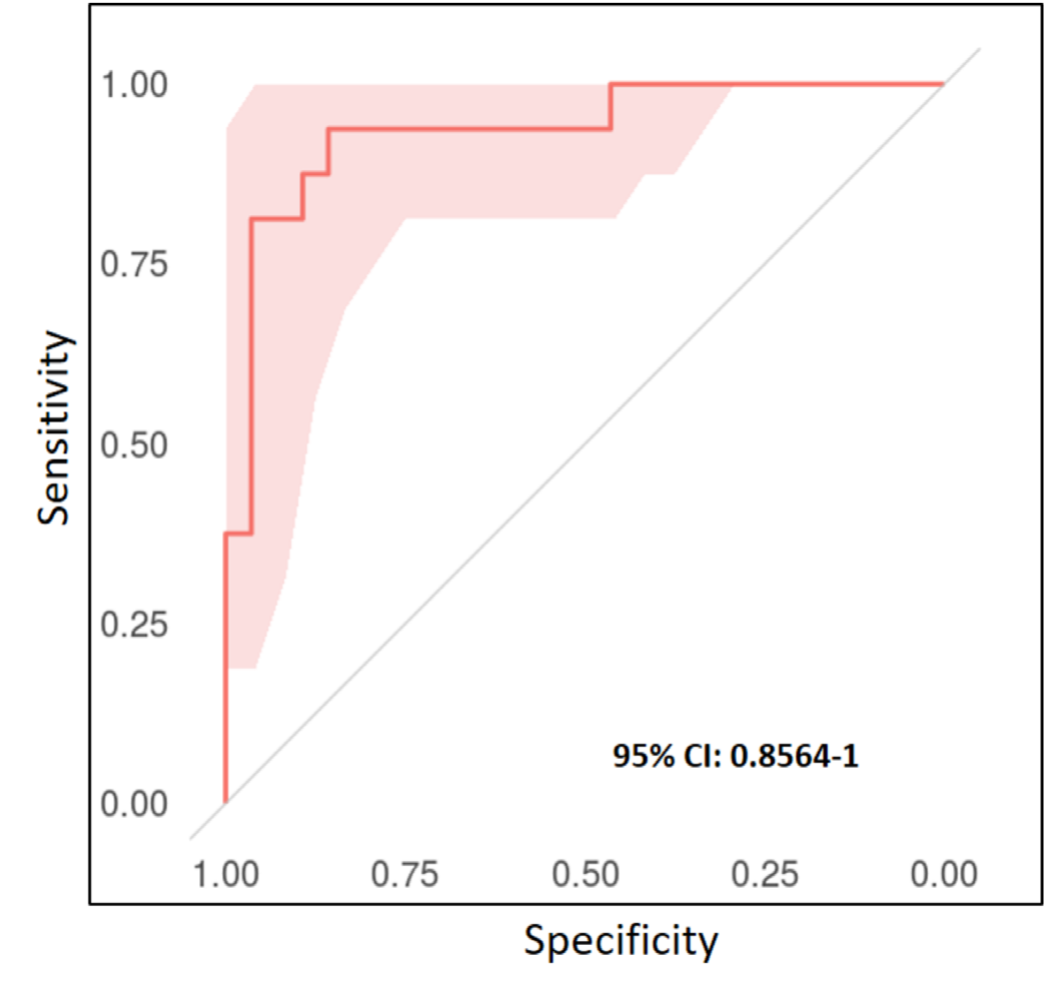
Slide-level receiver operating characteristic (ROC) curve with 95% confidence intervals of the ensemble of 5 models (region-correlation) trained on 1024 × 1024 patches.

Table 6 presents the performance metrics for slide-level classification. The best performance was obtained from the model (1024)-4 derived from independent patch classification, with the accuracy, sensitivity, specificity, precision, F1, and AUC being 0.864, 0.813, 0.893, 0.813, 0.813, and 0.940, respectively. The performance by the “five model ensemble” was close to that achieved by the best of the five individual models. Unlike the patch-level performances; however, the slide-level classifications derived from the patch classification with region correlation are not significantly superior to the counterparts that derived from independent patch classifications. Due to the limited number of slides for test (44 UM WSI), the 95% CIs were comparably wide (e.g. 0.881-1 and 0.856-1 for the ensembles of the independent case and region correlation case, respectively), as shown in Figures 6 and 7, and Table 6.

**Table 6.** Slide-Level Performances of the 5 Models (on 1024 × 1024 Patches) on the Test Set

**Independent**						
	**Model (1024)-1**	**Model (1024)-2**	**Model (1024)-3**	**Model (1024)-4**	**Model (1024)-5**	**Ensemble**
Accuracy	0.931	0.795	0.886	**0.864**	0.818	0.863
Sensitivity	**0.937**	0.875	0.875	0.813	0.750	0.875
Specificity	0.928	0.750	0.892	**0.893**	0.857	0.857
Precision	0.882	0.666	0.823	**0.813**	0.750	0.777
F1	0.909	0.756	0.848	**0.813**	0.750	0.823
AUC	0.953	0.915	0.928	0.940	0.915	0.944
**Region Correlation**						
	**Model (1024)-1**	**Model (1024)-2**	**Model (1024)-3**	**Model (1024)-4**	**Model (1024)-5**	**Ensemble**
Accuracy	0.886	0.840	0.840	**0.909**	0.863	0.886
Sensitivity	**0.875**	0.750	0.812	0.812	0.750	0.812
Specificity	0.892	0.892	0.857	**0.964**	0.928	0.928
Precision	0.823	0.800	0.764	**0.928**	0.857	0.866
F1	0.848	0.774	0.787	**0.866**	0.800	0.838
AUC	0.912	0.863	0.890	**0.939**	0.915	0.935

## Discussion

In this study, to the best of our knowledge, we demonstrate for the first time that a pilot DL model can be applied to predict nBAP1 expression in uveal melanoma from H&E-stained sections only, which is very difficult (if not impossible) by human pathologists. Although our model would benefit from a multicenter external validation study, our results demonstrate a “proof-of-concept” (i.e. it provides a feasible alternative to estimate nBAP1 status), and thereby the risk of UM metastasis. It is particularly beneficial for those laboratories without access to high quality IHC facilities or to genetic testing. In addition to independent patch classification, we further verified that region correlation was able to improve the performance of a patch-level classification.

Our results show that the developed models for patch classification, which were trained on different subsets of the whole training set, were able to achieve promising performances. Particularly the best model (1024)-4 by region correlation could achieve an AUC value of up to 0.90. For slide-level prediction, the AUC value derived from independent patch classification by the best model (model (1024)-4) was up to 0.94.

We further created an “ensemble” model by averaging the posterior probabilities of a patch of certain dimensions from the five trained models, and the related performance of the ensemble was best (or close to the best) when compared with the corresponding five models. Although it could not outperform the five models, the ensemble operation has practical relevance. That is, in the clinical “real-world” scenario, the “ground truth” (i.e. the nBAP1 status) of an individual slide or a tumor patch is not always available, and, therefore, it is not possible to determine which individual model would give the best prediction. By applying the ensemble model, it would guarantee the best result close to optimal.

Prognostication in UM usually entails the incorporation of clinical, histomorphological, and genetic parameters.^3^^,^^4^ The latter information may not be available in all ocular centers and hence prognostication for patients with UM is based predominantly on the American Joint Committee on Cancer (AJCC)/ tumor size, lymph nodes affected, and metastases (TNM) staging system (i.e. on clinical, anatomic, and morphologic parameters),^31^ However, direct manual analysis of digital histopathological images has proven feasible and efficient to predict and detect the related gene status of tumor cells, as a potential surrogate to both IHC and genetic testing.^14^ This, however, requires a large number of hours of repetitive work by pathologists, annotating slides to determine the “ground truth.”^32^^,^^33^ In recent years, there has been an appetite to apply AI-related techniques, especially DL, for the automated analysis of digital histopathology images. Data-driven approaches have resulted in an improvement in DL techniques with respect to their objectiveness, reproducibility, and accuracy, and have provided new insights into various pathological features, as indicated below. There are numerous studies using DL to assess features, such as cell/cytoplasm segmentation,^34^^,^^35^ detection of mitoses,^36^^–^^40^ tubules,^41^^,^^42^ nuclei, and nucleoli,^43^ and grading of cancers,^41^^,^^44^ etc. Yet, there are very few studies focused on using DL on the analysis of digital pathological images to indirectly predict gene or protein expression status (e.g. from a conventional stain, such as the H&E). To the best of our knowledge, Sun et al.^30^ is the first group that developed a DenseNet^24^ model to predict nBAP1 expression on BAP1-IHC stained UM patches. Although this work was groundbreaking in UM, a potential weakness of the authors’ study was the risk of “information leakage” caused by splitting patches from the same slide into both the training and test sets. In the current study, we have challenged the DL to greater levels (i.e. by asking it to predict the nBAP1 IHC result from an H&E-stained section), something that even for a well-trained and experienced histopathologist would be quite difficult to do. Indeed, when we applied the Sun et al. method to our dataset, we could demonstrate that our regional correlation method designed on H&E sections outperforms that based on the BAP-1 stained UM slide,^30^ and that the performance gain can be even up to 5% (from model (1024)-4).

One of the major concerns to develop a machine learning model is its functionality and applicability (i.e. generalization ability). A model with higher generalization ability should be able to achieve better predictions using other unseen broader datasets. To this end, in our study, we adopted various measures to design and train the models. The choice of the ResNet-18, which served as the first-stage classifier as well the feature extractor, was chosen, because ResNet-18 has comparably fewer parameters to train, and with extensive batch normalization layers, it could alleviate the issue of “over-fitting” to some extent.^45^ Further, data augmentation approaches were adopted to relieve the limitation of a relatively small dataset, which included color jitter, random cropping, and random spatial transformation, etc. To better verify the generalization ability of the developed models, the experiments were elaborately designed and used two digital scanning platforms. First, there were five models, instead of just one model, trained on different subsets of the training set using patches of certain dimension. The corresponding experimental results showed the robustness and the highly generalization ability of our developed methods, because the trained models all achieved good results on the test set. Second, we split the whole dataset into a training set and a test set in a slide-based way, to avoid information leakage, ensuring that patches from the same slide would not simultaneously appear in both sets. Last, we collected training and test slides separately from two scanners with different specifications, respectively. Therefore, variances in WSI were introduced among the two groups of data. In future work, we aim to validate the developed models on a wider range of external UM cases.

Despite the promising performances of all the trained models, there are still some aspects that can be refined to gain an even better performance of the proposed method. We have used 184 slides in total for training (*n* = 140) and testing (*n* = 44). The patches from the same slide are similar in tissue morphologies, pigments, etc., and thus these patches, although the total number is large, have limited “knowledge” from the perspective of a DL model. In other words, although the number of tumor patches from all the slides is more than enough, the whole dataset trialed is still relatively small. This also resulted in the wide range of 95% CI for slide-level classification. With a greater number of cases, we believe the developed model can achieve better performance. Hence a large multicenter validation study would be of value to externally validate and potentially revise our pilot model for ultimate application in routine laboratories. Such a larger study would also provide a broader spectrum of tumor size and shape, enabling improved training of the DL network on small UM. In our current study, the only clue utilized for the prediction of nBAP1 expression (and therefore prognostication) was the H&E-stained slides. Other well-known prognostic variables for UM, such as patient age and gender, were not considered in this work. In the future, we intend to develop a multivariable prognostic model, similar to (or a revision of) the Liverpool-developed prognostic algorithm, LUMPO3,^46^ which could incorporate the DL tool and its analysis of various morphological, IHC, and genetic parameters of UM.

In summary, through this work we showed that our new pilot DL techniques are able to effectively predict nBAP1 expression in UM using H&E-stained WSI images only. This work demonstrates a proof-of-concept of AI-related techniques for automated analysis of basic histological data, and could be translatable into routine laboratories. It is an important step toward an automated prediction of UM dissemination by applying digital pathology, as is already done in other cancer types.

## Supplementary Material

Supplement 1

Supplement 2
